# Hyaluronic Acid Improves Hydrogen Peroxide Modulatory Effects on Calcium Channel and Sodium-Potassium Pump in 4T1 Breast Cancer Cell Line

**DOI:** 10.1155/2020/8681349

**Published:** 2020-12-30

**Authors:** Ardeshir Abbasi, Nafiseh Pakravan, Zuhair Mohammad Hassan

**Affiliations:** ^1^Department of Immunology, Faculty of Medical Sciences, Tarbiat Modares University, Tehran, Iran; ^2^Department of Immunology, Medical School, Alborz University of Medical Sciences, Karaj, Iran

## Abstract

Maintaining homeostasis of ion concentrations is critical in cancer cells. Under hypoxia, the levels of channels and pumps in cancer cells are more active than normal cells suggesting ion channels as a suitable therapeutic target. One of the contemporary ways for cancer therapy is oxidative stress. However, the effective concentration of oxidative stress on tumor cells has been reported to be toxic for normal cells as well. In this study, we benefited from the modifying effects of hyaluronic acid (HA) on H_2_O_2_, as a free radical source, to make a gradual release of oxidative stress on cancer cells while preventing/decreasing damage to normal cells under normoxia and hypoxic conditions. To do so, we initially investigated the optimal concentration of HA antioxidant capacity by the DPPH test. In the next step, we found optimum H_2_O_2_ dose by treating the 4T1 breast cancer cell line with increasing concentrations (0, 10, 20, 50,100, 200, 500, and 1000 *μ*M) of H_2_O_2_ alone or H_2_O_2_ + HA (83%) for 24 hrs. The calcium channel and the sodium-potassium pumps were then evaluated by measuring the levels of calcium, sodium, and potassium ions using an atomic absorption flame spectrophotometer. The results revealed that treatment with H_2_O_2_ or H_2_O_2_+ HA led to an intracellular increase of calcium, sodium, and potassium in the normoxic and hypoxic circumstances in a dose-dependent manner. It is noteworthy that H_2_O_2_ + HA treatment had more favorable and controllable effects compared with H_2_O_2_ alone. Moreover, HA optimizes the antitumor effect of oxidative stress exerted by H_2_O_2_ making H_2_O_2_ + HA suitable for clinical use in cancer treatment along with chemotherapy.

## 1. Introduction

Nowadays, cancer therapy includes surgery, radiotherapy, chemotherapy, hormone therapy, and cytokine therapies. However, none of the methods present a complete recovery procedure and even can damage healthy cells/tissues of the body. This raises the need to understand more about the cancer biology and its cross-talk with the immune system whose regulation or activation can be manipulated to develop a new anticancer immunotherapy approach such as immune checkpoint blockade [1]. Metastatic cancers, such as breast cancer, are much more resistant to treatment, and despite the current improvement achieved in immunotherapeutic approaches, their treatment still remains a challenge. 4T1 cell line originates from mice triple-negative breast cancer with a high metastatic capacity to the lung [2].

It is known that a combination of internal and external factors are involved in the initiation, development, and progression of breast tumors [3, 4]. The maintenance of ion homeostasis is crucial for normal cell life. Several pieces of literature have reported that the role of elements such as calcium (Ca^2+^), sodium (Na^+^), and potassium (K^+^) in the proliferation, signaling pathways, and metastatic protection of cancer cells is undeniable. On this basis, the homeostasis of these elements is regarded as an important issue in tumor biology. For example, intracellular Ca^2+^ plays a prominent role in cell function involved by enzyme regulation, gene activation, proliferation, apoptosis, cell cycle, and production/performance of transmitters/sensors [5, 6]. Ca^2+^ signaling in cancer is associated with various types of processes in the tumor microenvironment, such as tumorigenesis, proliferation, migration, angiogenesis, and apoptosis escape [7, 8]. It has become evident that Ca^2+^ is required for activities such as cell motility, cell migration, and remodeling of cytoskeleton and extracellular matrix [9]. In addition, the Na^+^-K^+^ ATPase pump (NKA) as an integrated protein in the cell membrane can maintain the equilibrium Na^+^-K^+^ between the two sides of the cell membrane. Homeostasis of such an equilibrium is important in metabolic activities of the cell as well as other cellular processes such as proliferation, motility, and apoptosis [10].

There is a piece of considerable evidence demonstrating that the amount of NKA and Ca^2+^ channel is increased to exchange the ions at a higher level than that of the normal cells in cancer cells [11]. Consequently, it can be concluded that the NKA pump and Ca^2+^ channels are good targets for cancer therapy. Given the ability of oxidative stress in disrupting these pumps/channels, oxidative stress can be applied to induce tumor cell death. While ROS regulation at a moderate level is crucial to maintain homeostasis of normal cells to function, higher levels of ROS cause damage to them [12]. One of the most convenient sources of ROS is H_2_O_2_. Hydrogen peroxide plays vital roles in pathways which are involved in the regulation of cell proliferation, differentiation, migration, and apoptosis [13, 14]. It follows the importance of precise maintenance of ROS level which affects the flexibility of ion channels/pumps such as Ca^2+^ and Na^+^/K^+^ [12]. ROS can increase Na^+^ level in several ways including (a) changing in the voltage of the Na^+^ channel leading to Na^+^ accumulation in the cells [15] and (b) activating the Na^+^/H^+^ pump promoting Na^+^ into the cell [16]. In a simple word, channel disruption causes failure in cell energy production [17] or inhibition of Ca^2+^ extrusion. Additionally, numerous studies have reported dysregulated potassium channel expression in human cancer cases. However, knowledge about the redox modulation of K^+^ channel activity is limited. Oxidative stress potentially influences the expression of KV1.5 potassium channel expression in both physiological and pathological conditions [18]. Preclinical studies conducted on the effects of oxidative stress and free radicals on cancer cells have shown that oxidative stress at the administered concentrations might be a possible explanation for the substantial toxicity associated with the clinical management and cancer treatment. Therefore, new approaches are necessitated to target the NKA pump and Ca^2+^ channel using potent agents against cancer with no or least harm for the normal cells. It requires the utilization of reagents mediating the gradual release of free radicals. This would help us to optimize the therapeutic effects of oxidative stress. HA is one candidate to achieve this aim.

**Figure 1:**
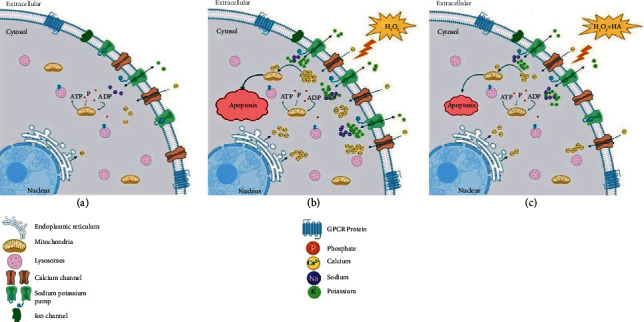
Schematic illustration of how H_2_O_2_ or H_2_O_2_ + HA functions on 4T1 cell ion channels/pumps. (a) The cell is normal and the channels function well to maintain the homeostasis of cell ions. (b) The membranous channels/pumps of cells exposed to H_2_O_2_ are disrupted, and the balance of ions is disrupted within the cell causing a severe imbalance due to an increase in intracellular Ca^2+^, K^+^, and Na^+^. Intracellular Ca^2+^ accumulation induces apoptosis in the cell. (c) HA softens oxidative effects of H_2_O_2_, modifies the harsh release of oxidants from H_2_O_2_, controls cell proliferation, and gently directs the cell toward apoptosis.

To exert and evaluate the effect of oxidative stress on Ca^2+^, K^+^, and Na^+^ concentrations in 4T1 tumor cells, we utilized H_2_O_2_ along with HA with the aim of suppressing Ca^2+^ channels and NKA pumping and inducing apoptosis tumor cells. HA along with H_2_O_2_ was used to protect normal cells from oxidative stress damages (see Figure 1).

## 2. Materials and Methods

### 2.1. Cell Culture

Mouse breast cancer cell line 4T1 was purchased from the Pasture Institute, Cell Bank of Iran (NCBI, Tehran, Iran). The cells were cultured in RPMI 1640 medium supplemented with 10% fetal bovine serum (FBS) and 1% glutamine (Sigma Aldrich), 100 IU/ml streptomycin, and 100 IU/ml penicillin at 37°C in 5% CO_2_-humidified atmosphere.

### 2.2. Induction of Normoxic or Hypoxic Condition and Treatments with H_2_O_2_ and H_2_O_2_ + HA

Normoxic (18% O_2_, 5% CO_2_) or hypoxic (1% O_2_, 5% CO_2_, and 94% N_2_) condition was established in two different incubators (Labotec C200, Germany) at 95% humidity. For all tests, 4T1 cancer cells were treated by increasing the concentrations of H_2_O_2_ (0, 10, 20, 50, 100, 200, 500, and 1000 *μ*M) or H_2_O_2_ + HA (0.83%) (0, 10, 20, 50, 100, 200, 500, and 1000 *μ*M and HA 0.83%) according to normoxic or hypoxic condition and incubated in two different incubators for 24 hrs.

### 2.3. Characteristic Hypoxic Model by Anti-PDL-1

To characterize the hypoxic condition, 4T1 cells were seeded in 12-well plates (1 × 10^5^ cells/well) in a regular growth medium and incubated in normoxic (18% O_2_, 5% CO_2_) and hypoxic (1% O_2_, 5% CO_2_, and 94% N_2_) conditions in two distinct incubators (Labotec C200, Germany) at 95% humidity overnight. Then, cells were collected, washed with phosphate-buffered saline (PBS), and re-suspended in 100 *μ*l PBS. Next, 5 *μ*l anti-PDL-1 mouse antibody conjugated with FITC was added to cells and incubated for 30 minutes at room temperature. Lastly, we added 400 *μ*l flow cytometry staining buffer and performed the flow cytometry, by flow cytometer (BD Biosciences, San Diego, CA, USA). The data were analyzed using FlowJo version 7.6.1.

### 2.4. Antioxidant Capacity of HA by DPPH Assay

The 2,2-diphenyl-1-picrylhydrazyl (DPPH) radical scavenging assay was performed according to the Blois method with some modifications [19]. Briefly, a 1.0 mM of DPPH radical solution in ethanol was prepared, and then, 1 ml of the solution was mixed with 3 ml of (0, 0.25, 0.5. 0.75, 1%) HA solutions. The absorbance was measured at 517 nm after 30 min. The proportion of DPPH radical scavenging was initially calculated as reported. All experiments were performed three times, and the average values were determined.

Determination of intracellular Ca^2+^, Na^+^, and K^+4^T1 cells was cultured in a six-well in the concentration of 1 × 10^6^ cells/well, incubated for 24 hrs under the normoxic or hypoxic conditions, and treated with H_2_O_2_ or H_2_O_2_ + HA for 24 hrs. At the end of the incubation time, the supernatant was discarded and the cells were trypsinized and centrifuged at 1500 rpm for 5 min. 1 × 10^6^ cells were collected and washed twice with Ca^2+^, Na^+^, and K^+^-free wash solution as previously described by Montrose in 1991 [20]. To prepare the solution, 130 mM tetramethylammonium (TMA) chloride (Merck, Germany, no. 822156), 1 mM MgSO4 (Merck, Germany, no.105886), 2 mM MgCl2 (Merck, Germany, no.874733), 0.1 mM EGTA (Merck, Germany, no. 99590-86-0), and 10 mM HEPES (Merck, Germany, no. 7365-45-9) were dissolved in ultrapure water, and pH was adjusted at 7.4 using TMA hydroxide (Merck, Germany, no. 75-59-2). Afterward, Ca^2+^, Na^+^, and K^+^ were determined using an atomic absorption flame spectrophotometer (GBC AA 932). The equipment was calibrated with standard solutions recommended by the manufacturer. All the samples were analyzed in triplicate.

### 2.5. Statistical Analysis

Statistical difference was determined by one-way and two-way ANOVA analysis of variance using GraphPad Prism 7.0 for Windows (GraphPad Software, Inc., San Diego, CA, USA). ^∗^*p* < 0.05, ^∗∗^*p* < 0.01, ^∗∗∗^*p* < 0.001, and ^∗∗∗∗^*p* < 0.0001 were considered as significance levels for all analyses performed. Data were presented as mean ± SD in triplicate experiments.

## 3. Results

### 3.1. Comparison of PD-L1 Level as Malignancy Marker in Hypoxic and Normoxic Conditions

**Figure 2:**
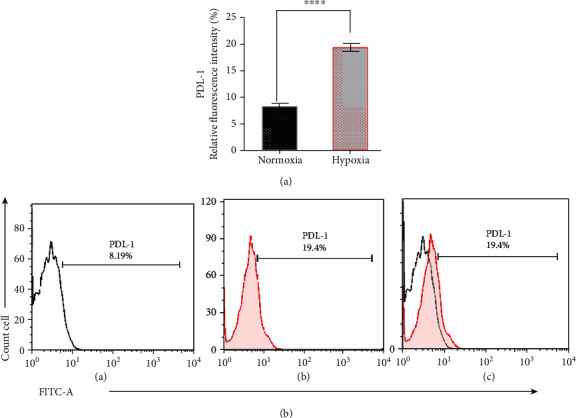
Characterization of the hypoxic condition by analysis of PDL-1 expression on 4T1 cancer cell membrane after 24 hrs of incubation (a). Different rate of PDL-1 expression on 4T1 cancer cell membrane under normoxic or hypoxic condition after 24 hrs of incubation (b). (A) Normoxia, (B) hypoxia, and (C) merge of (A) and (B).

To determine how hypoxic or normoxic condition affects 4T1 cell line, we used PD-L1 as a marker of malignancy. PD-L1 is one of those markers whose expression is increased under hypoxia conditions and enables tumors to escape from the adaptive immune system [21]. Flow cytometry analysis of 4T1 cells under the normoxic or hypoxic circumstances revealed that the PD-L1 surface expression under the hypoxic conditions significantly increased compared to 4T1 cells under the normoxic condition, as shown in Figure 2.

### 3.2. Evaluation of Optimizing Effect of HA on H_2_O_2_

**Figure 3:**
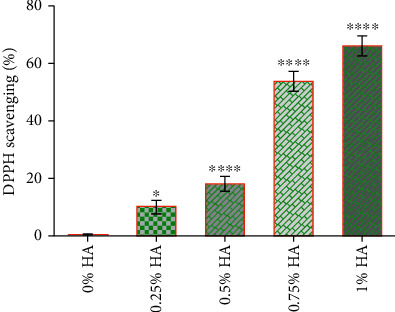
Scavenging of DPPH radical by hyaluronic acid: hyaluronic acid was prepared at concentrations of (0, 0.25, 0.5, 0.75, 1%), and then, the modifying capacity of hyaluronic acid on oxidative stress was investigated with the DPPH assay. Data are expressed as mean ± SD of three independent experiments; undisclosed SDs fall within respective symbols.

The optimizing effect of HA on H_2_O_2_ was evaluated using DPPH assay. The results indicated that HA optimized H_2_O_2_-mediated oxidative stress in a dose-dependent manner. However, HA at concentrations of 1% and 2% did not show a significant difference (see Figure 3). On this basis, effective concentrations were chosen for the subsequent experiments.

### 3.3. Ca^2+^ Level following Treatment with H_2_O_2_ or H_2_O_2_ + HA under Normoxic and Hypoxic Conditions

**Figure 4:**
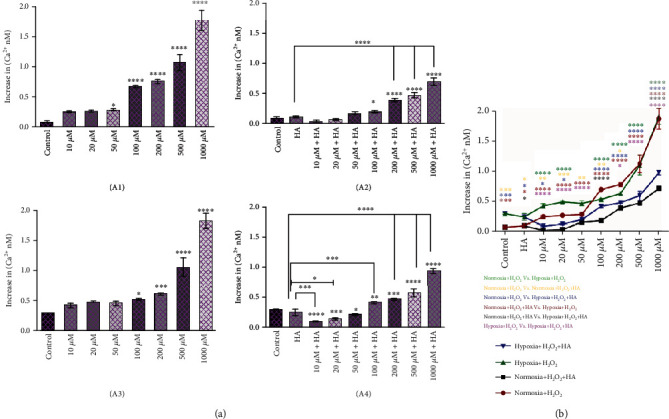
Effect of H_2_O_2_ or H_2_O_2_ + HA on the intracellular Ca^2+^ levels in breast cancer cells treated under normoxic or hypoxic conditions after 24 hrs of treatment: (a) (a1) Cells under normoxia and treated with H_2_O_2_. (a2) Cells under normoxia and treated withH_2_O_2_ + HA. (a3) Cells under hypoxia and treated with H_2_O_2_. (a4) Cells under hypoxia and treated with H_2_O_2_ + HA. Data are shown as mean ± SD (*n* = 3) (^∗^*p* < 0.05, ^∗∗^*p* < 0.01, ^∗∗∗^*p* < 0.001, and ^∗∗∗∗^*p* < 0.0001*vs.* respective control). (b) Comparison of intracellular Ca^2+^ levels in 4T1 cancer cells after 24 h of treatments with different concentrations in normoxia and hypoxia conditions determined by scratch assay. Data are shown as mean ± SD (*n* = 3) (^∗^*p* < 0.05, ^∗∗^*p* < 0.01, ^∗∗∗^*p* < 0.001, and ^∗∗∗∗^*p* < 0.0001*vs.* similar concentrations in different conditions. Each index is represented with assigned color in the legend).

Intracellular Ca^2+^ level in cancer cells is affected under the influence of microenvironmental conditions and treatments. Treatment with raising concentrations of H_2_O_2_ increased intracellular Ca^2+^ under normoxic conditions. Accordingly, treatment with H_2_O_2_ + HA also led to increased intracellular Ca^2+^ content in a dose-dependent manner though the extent of increase was about half that of the treatment with H_2_O_2_ alone (see Figure 4). Notably, the intracellular Ca^2+^ accumulation in H_2_O_2_ + HA-exposed cells had a gentler slope in the increment rate than that of the H_2_O_2_-treated cells in either normoxic or hypoxic conditions. In addition, the intracellular Ca^2+^ accumulation in hypoxic conditions is lower compared to the normoxic condition. This may be due to the adaptation of cancer cells with their specific hypoxic condition of the microenvironment, as demonstrated in Figure 4.

### 3.4. Treatment with H_2_O_2_ and H_2_O_2_ + HA Affects Na^+^ and K^+^ Levels under Normoxia and Hypoxia

**Figure 5:**
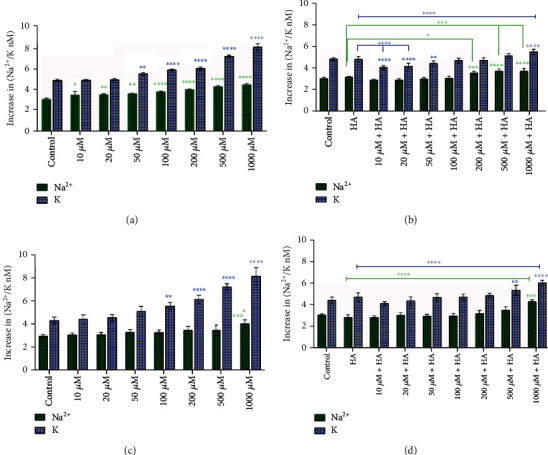
Changes in Na^+^ and K^+^ levels in 4T1 cancer cells treated with different concentrations of H_2_O_2_ or H_2_O_2_ + HA under normoxia and hypoxia for 24 hrs. (a) Cells under normoxia and treated with H_2_O_2_. (b) Cells under normoxia and treated with H_2_O_2_ + HA. (c) Cells under hypoxia and treated with H_2_O_2_. (d) Cells under hypoxia and treated with H_2_O_2_ + HA. Data are expressed as mean ± SD from three independent experiments (^∗^*p* < 0.05, ^∗∗^*p* < 0.01, ^∗∗∗^*p* < 0.001, and ^∗∗∗∗^*p* < 0.0001*vs*. respective control).

In order to investigate the NKA pump function, we examined the changes in intracellular Na^+^ and K^+^ levels. As shown in Figure 5, treatment of cancer cells with increasing concentration of H_2_O_2_ under normoxic conditions led to increment in intracellular K^+^ and Na^+^ levels as well. However, the rate of increase in K^+^ was far greater than in Na^+^. Instead, cancer cells treated with H_2_O_2_ + HA under normoxic conditions had lower intracellular K^+^ and Na^+^ accumulation levels.

The results under hypoxic conditions were similar to those of normoxia. Treatment of cancer cells with H_2_O_2_ increased the intracellular Na^+^ and K^+^ levels in a dose-dependent manner. In contrast, treatment of the cells with H_2_O_2_ + HA caused milder changes compared to the treatment with H_2_O_2_.

Overall, the comparison of intracellular Na^+^ and K^+^ in cancer cells under hypoxia and normoxia showed that the cells under hypoxia were more resistant to H_2_O_2_ and H_2_O_2_ + HA treatment than normoxia (see Figure 5).

## 4. Discussion

Hypoxia is one of the important features of solid tumors. Several mechanisms are presumed to involve in the development of hypoxic conditions within the tumor foci. They include limited perfusion and/or delivery of O_2_ delivery [22]. Tumor cells adapt to persistent hypoxic and harsh conditions and consequently become more invasive and metastatic. This is evidenced by the treatment resistance of tumor cells to a number of anticancer agents. Normal cells typically die in hypoxic conditions while tumor cells adapt to hostile hypoxic microenvironments and remain viable due to hypoxia-mediated proteomic and genomic changes within tumor cells [23].

Despite significant advances toward cancer biology in the last decade, the survival rate of cancer patients has remained insignificant. The approximate failure of the treatment procedure and also the increased cancer cell resistance might be due to several reasons such as (1) hypoxic condition in a cancer microenvironment that plays an essential role in tumor survival and resistance, (2) increasing the number of pumps/channels on cancer cells to bypass the apoptotic pathways, and (3) inefficiency of anticancer drugs because of the host condition and side effects. The development, survival, and progression of tumors depend on a combination of internal and external factors of which is the balance of ions [3, 4]. Maintaining the ionic homeostasis and optimal electrical potential is vital for survival in cell biological settings. On this basis, a new approach of drug designation has been developed focusing on ion transport pumps/channels in cell membranes to remove cancer cells. Oxidative stress is one of the novel approaches for cancer therapy. In this regard, many points have not yet been addressed and examined about the pathophysiological effects. This study was based on the premise that ROS kills cancer cells by disrupting the ion transport pumps/channels in cancer cell membranes and eventually induce apoptosis and death in cancer cells.

There are currently therapeutic approaches known as radiotherapy and photodynamic therapy that function based on the production of oxygen radicals within cancer cells [24]. Notably, pretreatment with H_2_O_2_ in radiotherapy can optimize the cancer microenvironment and improve the efficiency of clinical outcomes [25]. In this approach, ROS production is the mechanism by which apoptosis is induced in tumour cells [24, 26–29]. Given the point that cancer cells are highly dependent on ROS homeostasis compared to normal cells, they are more sensitive to further variation in their redox homeostasis compared to normal cells. This is because ROS can act as a double-edged sword and affect tumour cells more than normal cells [30]. Presumably, H_2_O_2_ kills tumour cells via direct induction of ROS production in cancer cells and inhibition of antioxidant machinery as a defence mechanism of cancer cells. The applied concentration of H_2_O_2_ to kill tumour cells should be optimized depending on the antioxidant potential of cancer cells [31, 32]. Previous studies utilized H_2_O_2_ as a radiosensitizer material and even H_2_O_2_ along with sodium hyaluronate have further been used as a radiosensitizer. These approaches are targeted to inactivate cell antioxidant enzymes and produce oxygen from H_2_O_2_ to decrease hypoxia [33, 34]. Given the important role of oxygen deprivation in tumour adaptation and development, it has been proposed that oxygenation can restore health by destroying cancer cells. Supporters of oxygen therapy claim that low levels of oxygen enable tumour cells to adapt and thrive. Accordingly, oxygenation of tumour cells interferes with their proteomic and genomic changes and destroys them. Hydrogen peroxide is one of the oxygenating agents. The initial idea for the medical use of hydrogen peroxide dates back to decades ago when antineoplastic hydrogen peroxide was observed [35–38]. However, claims that hydrogen peroxide therapy can increase cellular levels of oxygen have been a matter of debate. Since hydrogen peroxide decomposition and the type of product mainly depend on the microenvironment [39], it may produce OH radical or water and oxygen. On the other hand, HA is one of the chemicals which facilitate the production of oxygen from hydrogen peroxide. Accordingly, it has been shown that hyaluronic acid, commonly used as a drug delivery vehicle [40], is capable of increasing the toxic effects of the oxidative stress mediated by H_2_O_2_ on tumour cells [41]. This is consistent with the fact that CD44, as major receptor HA, is abundantly expressed on cancer stem cells [42] which facilitates targeting of toxic effects of H_2_O_2_ on cancer cells. In this study, we utilized hydrogen peroxide along with HA to investigate how it affects 4T1 cell line *in vitro*. H_2_O_2_ could alternatively be a source of free radicals to exert oxidative stress or could be an oxygen-producing source on cancer cells.

Hydrogen peroxide is one of the inhibitors of PI3K/AKT and the PTEN pathway [18]. Cancer cells adapted themselves to hypoxic conditions to escape from the immune response against cancer. Some of these escape mechanisms include the increase in PDL-1 receptor and elevation of NKA pump and Ca^2+^ channel levels involved in cellular ion regulation [11]. In this study, the simulation of hypoxic conditions in vivo was confirmed in cancer cells by increasing the PDL-1 expression. Previous reports have shown that Ca^2+^ plays a prominent role in tumor proliferation, angiogenesis, invasion, and apoptosis by remodeling of cytoskeleton and extracellular matrix [7, 8, 43]. Ca^2+^ homeostasis is regulated directly and indirectly by different molecules [7, 26, 28]. ROS disrupts these molecules, and subsequently, the membrane channels lose their regulatory function. As a result, disruption of these channels and related molecules changes intracellular Ca^2+^ level, increases Ca^2+^ uptake by mitochondria, and triggers apoptotic pathways leading to cancer cell death. Our results also showed that treatment of cancer cells with H_2_O_2_ under normoxic and hypoxic conditions increased intracellular Ca^2+^ level suggesting that the Ca^2+^ channels were disrupted. Interestingly, HA softened the H_2_O_2_ effect and made a gradual release of free radicals under both hypoxic and normoxic conditions.

ROS inhibits and disrupts the NKA pump and increases the Na^+^ levels in the cell in several ways including (1) changes the voltage of the NKA pump [15], (2) activates the NKA pump and releases Na^+^ into the cell [44], (3) degrades NKA proteasomal complex resulting in reduced expression of the pump at the membrane surface [45], and (4) activates protein kinase C (PK C) which induces NKA phosphorylation and membrane depolarization [46, 47] all of which lead to cell death. However, knowledge about the redox modulation of the K^+^ channel activity is limited. It has been shown that an increase in calcium can alter the K^+^ channels and increase K^+^ in the cell. Additionally, current data on the effect of oxidative stress on Ca^2+^-activated K^+^ channel (BK_Ca_) activity in vascular smooth muscle cells suggest that O_2_^–^ and H_2_O_2_ enhance the BK_Ca_ channel activity [48]. It has also been shown that oxidative stress (O_2_^–^ and H_2_O_2_) increases the K_ATP_ channel activity in cardiac myocyte vasculature [49]. Moreover, enhanced ATP-dependent K^+^ channel activity by H_2_O_2_ has also been observed in rabbit airway smooth muscle [50]. Our study showed that treatment of cancer cells with H_2_O_2_ under normoxia or hypoxia led to an increased intracellular Na^+^ and K^+^ in a dose-dependent manner indicating impaired NKA pumping. In contrast, treatment of cells with H_2_O_2_ + HA made these changes gradual. This again confirmed the modifying effects of HA on the oxidative stress capacity exerted by H_2_O_2_ consistent with previous reports in other settings [51]. Unique HA properties include biocompatibility, nontoxicity, and biodegradability which make it suitable to be applied in biomedical applications [52, 53].

Previous studies supported that H_2_O_2_ increases cell death induced by inhibition of PKC*ε*, PI3K, pAKT, pJAK-2, and pSTAT3 [54]. H_2_O_2_ also prevents the interaction of AP-1, NF-*κ*B, and STAT3 transcription factors with DNA [55, 56]. Our results on the effect of H_2_O_2_ on the NKA pump and Ca^2+^ channels can also be added to the above list as the cause of apoptosis induction by H_2_O_2_ in cancer cells.

Our results demonstrated that the control cells showed higher K^+^ and lower Na^+^ concentrations which is supported by previous studies. Increasing evidence suggests that ion channels and pumps not only regulate membrane potential, ion homeostasis, and electric signaling in excitable cells but also play important roles in cell proliferation, migration, apoptosis, and differentiation [57]. In addition, recent analysis has revealed complex interconnections between oncogenic activity, ion channels, hypoxia signaling, and metabolic pathways that are dysregulated in cancerous cells. Normal and tumor cells frequently express distinct pump isoforms. Several changes in the expression of NKA have been observed in cancer cells, such as elevation of activity during the transformation of malignant cells. Roles in cell survival, proliferation, adhesion, and migration have also been described [58–60]. Alterations in K^+^ channel subtypes have been confirmed in breast and colon cancer [61]. To maintain the homeostasis of cancer cells, these subunits increase and in some cases decrease; for example, the K2P channel KCNK9 is overexpressed in breast and lung cancer [62]. Recent work has demonstrated that oncogenic stress increases the KCNA1 expression and promotes its relocation from the cytoplasm to the plasma membrane, which is required for oncogene-induced senescence. Ectopic expression of KCNA1 inhibits the RAS-induced transformation, which is related to decreased aggressiveness in breast cancer [63]. In this context, variations in the expression of the different subunits of the enzyme compared to the normal tissues have also been described. On this basis, cancer has also been named as a channelopathy. Collectively, based on our knowledge, the differences in the expression, function, and activity of ion channels and pumps in cancer cells depend on different factors. These include types of channels, the differences in pump and ion channel isoforms on cancer cells, the type and class of cancer, the metastasis and invasiveness degree, the stage of cancer, and the duration of hypoxia.

We previously evaluated the effect of H_2_O_2_ or H_2_O_2_ + HA on 4T1 cancer cells and demonstrated that treatment of 4T1 cells with concentrations higher than 100 *μ*M of H_2_O_2_ or 200 *μ*M of H_2_O_2_ + HA leads to decreased survival, increased apoptosis, cell cycle disturbance, and decreased expression of MMP2, MMP9, and VEGF genes [26].

Overall, numerous preclinical studies have been done on the effects of oxidative stress on cancer cells with considerations on the dose as oxidative stress at some concentrations may be of higher toxicity and side effects [17]. So finding a way to get this problem under control is essential. In this study, we used HA to overcome toxic and side effects with optimal results. As a result, the effects of H_2_O_2_ combined with HA may provide us the opportunity to develop and modify cancer treatment using oxidative stress.

## 5. Conclusion

Our finding suggests considering the inactivation of the NKA pump and Ca^2+^ channels and triggering of cancer cell apoptosis when using chemotherapeutic agents containing oxidative stress. This study also gives an initial image on the rate and ratio of H_2_O_2_ plus HA usage to put control on our targeted selective and effective oxidative stress therapy.

## Data Availability

The data used to support the findings of this study are included within the article.
